# Pleural Fluid Has Pro-Growth Biological Properties Which Enable Cancer Cell Proliferation

**DOI:** 10.3389/fonc.2021.658395

**Published:** 2021-04-28

**Authors:** Rachelle Asciak, Nikolaos I. Kanellakis, Xuan Yao, Megat Abd Hamid, Rachel M. Mercer, Maged Hassan, Eihab O. Bedawi, Melissa Dobson, Peter Fsadni, Stephen Montefort, Tao Dong, Najib M. Rahman, Ioannis Psallidas

**Affiliations:** ^1^ Oxford Centre for Respiratory Medicine, Churchill Hospital, Oxford University Hospitals NHS Foundation Trust, Oxford, United Kingdom; ^2^ Laboratory of Pleural and Lung Cancer Translational Research, Nuffield Department of Medicine, University of Oxford, Oxford, United Kingdom; ^3^ Mater Dei Hospital, Msida, Malta; ^4^ National Institute for Health Research Oxford Biomedical Research Centre, University of Oxford, Oxford, United Kingdom; ^5^ Chinese Academy of Medical Sciences Oxford Institute, Nuffield Department of Medicine, University of Oxford, Oxford, United Kingdom; ^6^ MRC Human Immunology Unit, MRC Weatherall Institute of Molecular Medicine, University of Oxford, Oxford, United Kingdom; ^7^ Oxford Respiratory Trials Unit, Nuffield Department of Medicine, University of Oxford, Oxford, United Kingdom; ^8^ Research and Early Development, Respiratory & Immunology, AstraZeneca, Cambridge, United Kingdom

**Keywords:** pleural fluid, malignant pleural effusion (MPE), pleural metastases, malignant pleural mesothelioma, pleural cancer

## Abstract

**Objectives:**

Patients with malignant pleural mesothelioma (MPM) or pleural metastases often present with malignant pleural effusion (MPE). This study aimed to analyze the effect of pleural fluid on cancer cells.

**Materials and Methods:**

Established patient-derived cancer cell cultures derived from MPE (MPM, breast carcinoma, lung adenocarcinoma) were seeded in 100% pleural fluid (exudate MPM MPE, transudate MPE, non-MPE transudate fluid) and proliferation was monitored. In addition, the establishment of new MPM cell cultures, derived from MPE specimens, was attempted by seeding the cells in 100% MPE fluid.

**Results:**

All established cancer cell cultures proliferated with similar growth rates in the different types of pleural fluid. Primary MPM cell culture success was similar with MPE fluid as with full culture medium.

**Conclusions:**

Pleural fluid alone is adequate for cancer cell proliferation *in vitro*, regardless of the source of pleural fluid. These results support the hypothesis that pleural fluid has important pro-growth biological properties, but the mechanisms for this effect are unclear and likely not malignant effusion specific.

## Introduction

Malignant pleural effusion (MPE) is a common complication which affects patients with pleural metastases, or malignant pleural mesothelioma (MPM) (1, 2). The incidence of MPE is rising worldwide, driven by the increasing prevalence of cancer and advances in cancer management and treatment (3–5). The development of MPE has been linked with poor survival outcomes (5). MPE is an established prognostic factor for shorter life expectancy for patients with lung cancer (6). Treatment options include observation, drainage and pleurodesis (5). Currently, treatment remains palliative and is mainly focused on symptom relief. Therefore, it is recommended to only drain symptomatic MPE (2, 5, 7), however, there is some early pre-clinical evidence that MPE may not simply be a bystander. It has been shown that MPE may have biological properties that contribute to MPM proliferation and promote resistance to chemotherapy (8, 9).

MPE is a protein-rich fluid including growth factors and cytokines, with pro-inflammatory, oncogenic and angiogenic properties such as vascular endothelial growth factor (VEGF) (10, 11), and immunosuppressive such as IL-10 (12). This suggests the hypothesis that MPE fluid provides a nutrient-rich microenvironment to support tumor growth, while suppressing anti-tumor immune activity.

Laboratory data support this view - Cheah et al. reported that MPM cells exposed to 30% MPM MPE fluid *in vitro* had increased cell viability compared to cells exposed to serum-free and serum-enriched medium. In addition, MPM cell death was significantly less likely after exposure to cisplatin/pemetrexed combination in the presence of 30% MPM MPE fluid, than cells that were only exposed to the chemotherapy (8). Although this data provides early evidence of the potential biological properties of pleural fluid the results have not been replicated and there were some experimental limitations (use of control and 30% MPE fluid does not necessarily reflect the *in vivo* pleural effusion environment).

Patient-derived cancer cell cultures are a faithful laboratory model with the potency to recapitulate the biology of the disease (13–15). Patient-derived MPM cancer cell cultures from MPE samples reflect tumor and patient heterogeneity and closely resemble native tumor specimens (16). There is a need to further understand the role of pleural fluid. Use of 100% pleural fluid in experimental models may bridge some of the gap between *in vitro* and *in vivo* settings, potentially providing a closer representative of cancer cells *in vivo* which are bathed in pleural fluid. This study aimed to analyze whether pleural fluid alone is adequate for cancer cell proliferation *in vitro*.

## Materials and Methods

### Study Approval

Pleural fluid samples were collected from patients who gave consent for inclusion in the Oxford Radcliffe Pleural Biobank (Research ethics committee: South Central Oxford C 09/H0606/5+5). This project was approved by the Oxford Radcliffe Biobank Central University Research Ethics Committee (CUREC) number: 19/A107. Pleural Biobank is a prospective collection of clinical specimens derived from patients presenting with pleural effusion of varied diagnoses.

### Pleural Fluid Specimen Collection

Pleural fluid (50ml) was collected from patients with MPM who underwent pleural procedures as part of their standard medical care. The fluid was collected in sterile 50ml conical centrifuge tubes and processed for cell culture within 2-3 hours of sampling. The protocol used for cancer cell culture is described elsewhere (16). When pleural fluid was used as culture medium in the experiments, mostly fresh pleural fluid (kept in refrigerator at 3-4**°**C for up to 3 days) was used, but when this was not available, freshly-thawed-from-frozen unfiltered pleural fluid samples were used.

### Seeding of Cells From Established Patient-Derived Cancer Cell Cultures in MPM Exudate MPE Fluid

1. The cells from established patient-derived MPM cell culture (MESO-163, epithelioid MPM) were seeded at 100,000 cells per well in a 6-well plate, in 2ml of MPM MPE pleural fluid.
2. The cells were incubated at 37**°**C and 5% CO_2_.
3. Serial live images of the cells within the well were taken daily to monitor growth and proliferation.
4. The pleural fluid was refreshed every 48 hours.

### Seeding of Cells From Established Patient-Derived Cancer Cell Cultures in Any Pleural Fluid

MPM cells from established patient-derived MPM cell cultures [MESO-163 (epithelioid MPM), MESO-024 (biphasic MPM), MESO-027 (epithelioid MPM), MESO-031 (epithelioid MPM) were seeded at 20,000 cells per well in a 96-well plate, in starvation medium (Dulbecco’s modified eagle medium, Sigma-Aldrich^®^, D5671] for 12 hours before starvation medium was changed to 100μl of exudative MPM MPE fluid (biphasic MPM MPE fluid, with total fluid protein 39 g/l, glucose 2.3 mmol/l, LDH 679 IntUnit/l) or transudate MPE fluid (metastatic lung adenocarcinoma MPE (cytology positive for adenocarcinoma) with total fluid protein 8 g/l, glucose 5.7 mmol/l, LDH 190 IntUnit/l). There were at least 6 replicates for each cell culture at each time point. The pleural fluid was refreshed every 24 hours. Cell viability was assessed at 4, 8, 12, 24 and 48 hours using CellTiter-Glo^®^. A well-characterized, commercially available biphasic MPM cell line CRL-2081(MSTO-211H)™ (derived from human MPE fluid) was purchased from ATCC^®^ and used as a control.

This experiment was then repeated, this time seeding cells directly in pleural fluid without using starvation medium, with patient-derived MPM [MESO-163 (epithelioid MPM), MESO-174 (biphasic MPM), MESO-024 (biphasic MPM), MESO-027 (epithelioid MPM)], breast cancer (BRST-156) and lung adenocarcinoma (LNG-183) cell cultures, and with exudate and transudate MPE fluid, as well as non-MPE pleural fluid from a patient with heart failure-related pleural effusion (total fluid protein 25 g/l, glucose 5.7 mmol/l, LDH 97 IntUnit/l) (minimum of 3 replicates per cell culture). Cells were incubated in an Incucyte^®^ machine, and regular live images of the wells were obtained. For all experiments, cells were incubated at 37°C and 5% CO_2_.

### Calculation of Cell Size

Using Fiji (ImageJ) (17) version 2.0, the area of a sample of 3 cells from each image was calculated and a mean of the sizes obtained. This enabled comparison of size of the cells seeded in different pleural fluid types.

### Calculation of Cell Confluence

Pleural fluid was observed to have a tendency to adopt a gel-like texture *in vitro* (**Supplementary Figure 1**). Pleural fluid viscosity may lead to overestimation of the percentage confluence if this were to be calculated automatically, with viscous areas and fibrin strands being erroneously included as ‘area covered by cells’. To avoid this, the percentage confluence was calculated by measuring the pixels in the area of the well covered by cells, and expressing it as a percentage of the pixels in the total area of the well in the image (18). This allowed manual setting of the threshold for each image, ensuring that only the area of the well covered by cells was included when calculating percentage confluence (**Supplementary Figure 2**).

### Starting a New Cell Culture Without Using Culture Medium: Primary MPM Cell Culture in MPE Fluid, With Primary Culture in Full Culture Medium as a Control

Cells from MPM MPE fluid were seeded in full culture medium (Supplementary data) as per standard method used in our laboratory for primary cell culture, and in parallel, half the cells from the same MPE fluid sample were seeded in matched MPE fluid from the same patient instead of in full medium. The associated pleural fluid cytology result as reported by the clinical histopathologist as part of the patient’s routine clinical care was collected from the medical records. The method used was as follows:

1. Pleural fluid samples from patients diagnosed with MPM were centrifuged at 800G for 10 minutes and the supernatant was aspirated and saved for later use.
2. The sedimented cells were resuspended in 3-5ml of red blood cell lysis solution (Qiagen^®^) and cells were kept at room temperature for 5 minutes.
3. The sample was then centrifuged again at 500G for 5 minutes. The supernatant was aspirated and discarded, and the pelleted cells washed with phosphate buffered saline (Sigma-Aldrich^®^ MFCD00121855).
4. The sample was centrifuged one last time at 500G for 5 minutes. The pelleted cells were resuspended in 1ml of the MPE fluid saved from step 1 (or in 1ml full medium for the control), and then transferred to a cell culture treated plate with a further 9ml MPE fluid (or in 9ml full medium for the control) in it, and incubated at 37°C and 5% CO_2_.
5. The MPE fluid (or full medium for the control) was refreshed every 48 hours, and the plates were monitored regularly under a light microscope.
6. The cells were allowed proliferate until >90% confluence. The cells were then split, and about 70% of the cells were transferred to a new culture dish this time in full medium.

### Imaging

The serial live images of the cells within the wells were taken using ZEISS Axiocam 506 mono, to monitor growth and proliferation. The percentage confluence was calculated from the images using Fiji (ImageJ) (17) version 2.0.

### Statistics

Kruskal-Wallis test was used to compare mean size of cells seeded in the different pleural fluid types. GraphPad PRISM version 8.3.0 (GraphPad Software, San Diego, California USA) was used for the growth curves and the statistics.

## Results

### Cell Cultures Used in This Study

**Table 1** shows the baseline demographics of the patients the cell cultures used in this study were derived from.

**Table 1:** Shows the baselines demographics of the patients from whom the cell cultures used were derived from.

Cell culture	Patient age at fluid sampling	Patient gender	Histological diagnosis
MESO-163	63	F	Epithelioid MPM
MESO-174	69	M	Biphasic MPM
MESO-024	84	M	Biphasic MPM
MESO-027	56	M	Epithelioid MPM
MESO-031	78	M	Epithelioid MPM
BRST-156	NA	F	Breast carcinoma
LNG-183	NA	M	Lung adenocarcinoma

F, female; M, male; MPM, malignant pleural mesothelioma; NA, data not available.

### Cells From Established MPM Cell Cultures Proliferate in MPE Fluid Alone

**Figure 1:**
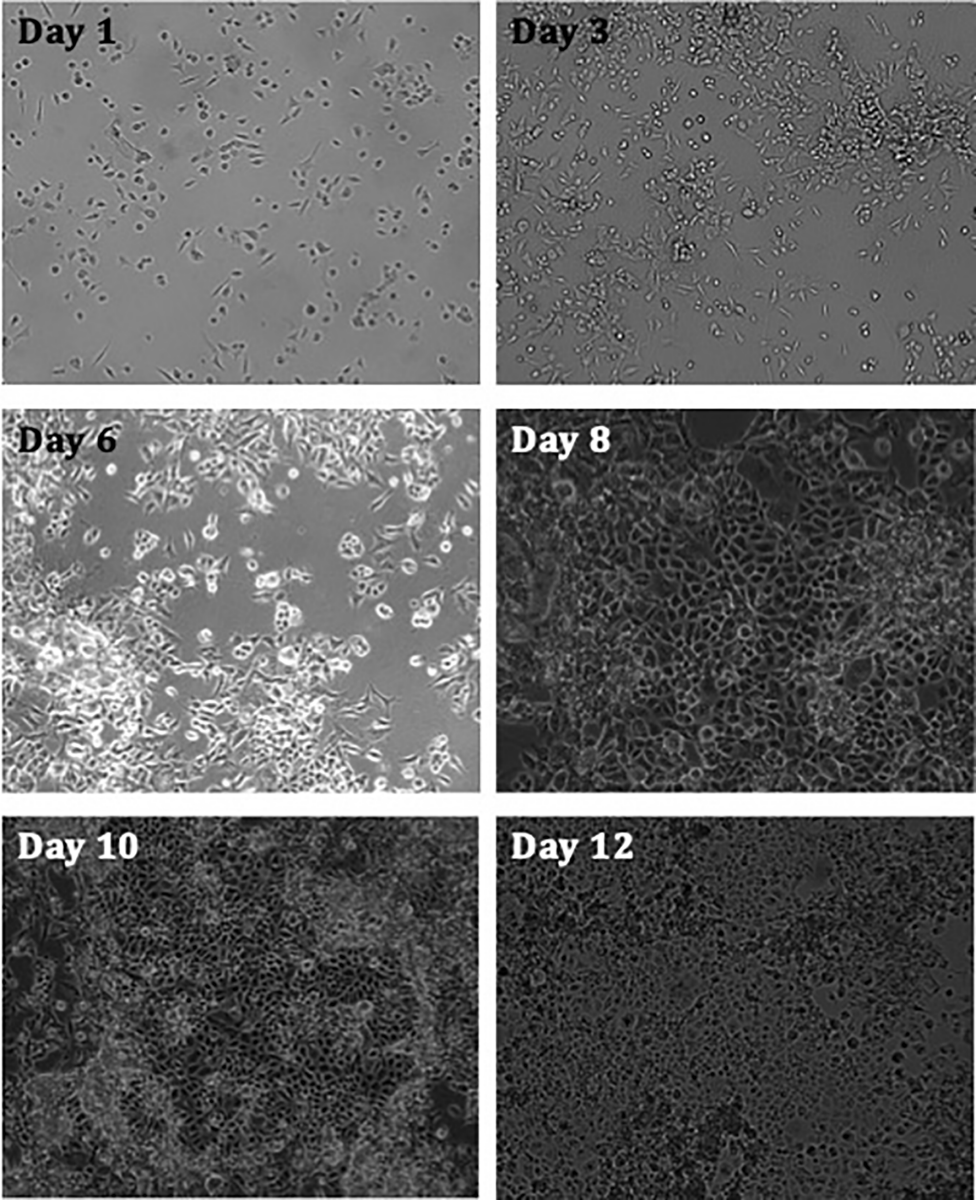
Images taken of MPM cell culture MESO-163 (epithelioid MPM) seeded in 100% exudate MPM MPE fluid only, at 100,000 cells per well in a 6-well plate. The images were taken on days 1-12 after seeding, and were taken at 10x magnification [ZEISS Axiocam 506 mono]. The cells show increased levels of confluency with time. *MPE, malignant pleural effusion; MPM, malignant pleural mesothelioma*.

The hypothesis that MPE fluid has biological properties was explored by first assessing whether cells from established MPM cell culture proliferate *in vitro*, obtaining nutrients solely from pleural fluid. Results revealed that the cells show increased levels of confluency with time (**Figure 1**).

### Cells From Cancer Cell Cultures Proliferate *In Vitro* in Exudate and Transudate MPE Fluid, as Well as in Heart Failure Transudate Pleural Fluid

**Figure 2:**
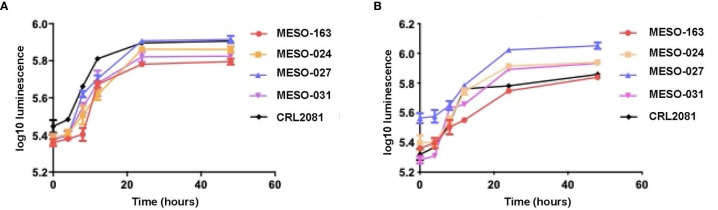
The growth curves with mean and 95% confidence intervals for each time point, obtained after 20,000 MPM cells per well (96-well plate) were seeded in starvation medium for 12 hours, then starvation medium was replaced with **(A)** exudate MPM MPE fluid and **(B)** transudate MPE fluid from a patient with lung adenocarcinoma MPE (right sided graph). Cell viability was measured at 4, 8, 12, 24 and 48 hours using CellTiter-Glo^®^. *Cell cultures: MESO-163 (epithelioid MPM), MESO-024 (biphasic MPM), MESO-027 (epithelioid MPM), MESO-031 (epithelioid MPM); CRL2081(MSTO-211H)™ - a well-characterized, commercially available biphasic MPM cell line (derived from human MPE fluid) used as a control*.

Subsequently, cells were seeded in pleural fluid in 96-well plates, and comparison of MPM cells’ growth in exudative MPM MPE fluid and transudative MPE fluid showed similar growth rates (**Figure 2**).

**Figure 3:**
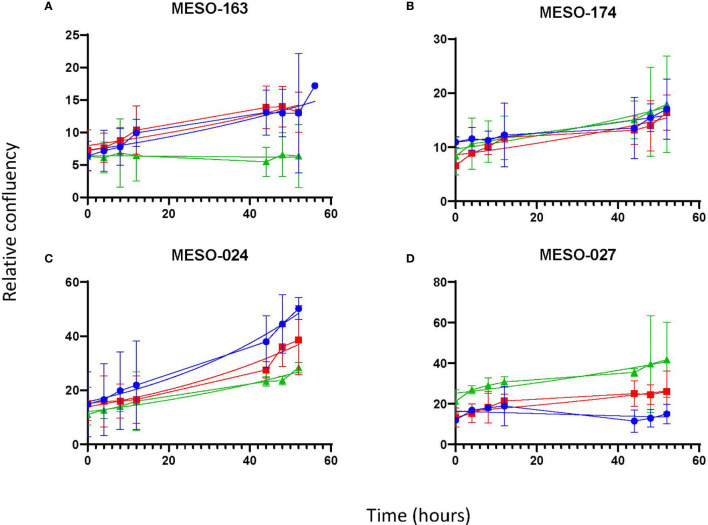
The growth curves for cells from MPM cell cultures *in vitro*, seeded directly in pleural fluid. The curves show the mean and 95% confidence intervals, and a trendline for non-linear fit. *Cell cultures:* **(A)** *MESO-163 (epithelioid MPM)*, **(B)** *MESO-174 (biphasic MPM)*, **(C)** *MESO-024 (biphasic MPM)*, and **(D)** *MESO-027 (epithelioid MPM). HF, heart failure; MPE, malignant pleural effusion; MPM, malignant pleural mesothelioma*.

**Figure 4:**
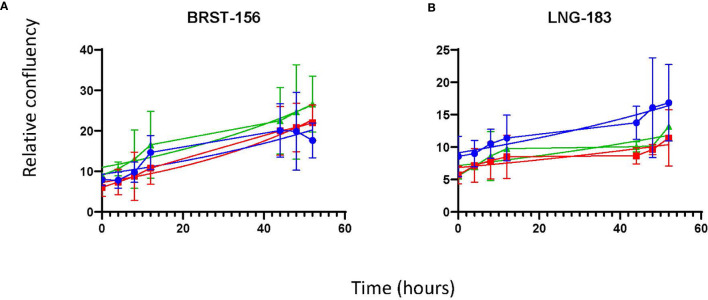
The growth curves for non-MPM cell cultures *in vitro*, seeded directly in pleural fluid. The curves show the mean and 95% confidence intervals, and a trendline for non-linear fit. *Cells cultures:* **(A)** *BRST-156 (breast carcinoma)*, and **(B)** *LNG-183 (lung adenocarcinoma). HF, heart failure; MPE, malignant pleural effusion*.

Once it was clear that MPM cells were able to proliferate *in vitro* in 100% MPE fluid, the latter experiment was repeated with cells from four MPM cell cultures [MESO-163 (epithelioid), MESO-174 (biphasic), MESO-024 (biphasic), MESO-027 (epithelioid)] to compare exudate and transudate MPE fluid, and transudate non-MPE pleural fluid (**Figure 3**). Breast carcinoma and lung adenocarcinoma cells also proliferated *in vitro* in 100% pleural fluid alone (**Figure 4**). For cell cultures MESO-163 and MESO-027, there was decreased proliferation with transudate MPE and with non-MPE heart failure transudate respectively, when compared to the proliferation with other types of pleural fluid tested. However, there was no clear decreased proliferation with any one pleural fluid type across all cell cultures tested.

There was no significant difference in cell morphology noted, and neither was there significant difference in size of cells seeded in the different types of pleural fluid (**Supplementary Figure 3**).

### Primary Culture of MPM Cells Can Be Achieved Using MPE Fluid Alone as Culture Medium

In order to further assess the biological properties of pleural fluid, primary MPM cell culture in MPE fluid was attempted in 6 MPM MPE fluid samples, with concurrent primary cell culture in the standard *in vitro* cell culture medium (DMEM enriched with 10%FBS) as a control. In 3 (3/6, 50%), cell culture was successful in both MPE fluid and cell culture medium, in 1/6 (16.7%) cell culture was unsuccessful from the start in cell culture medium and unsuccessful beyond passage 1 in pleural fluid, and 2 (2/6, 33.3%) attempts were unsuccessful in both MPE fluid and cell culture medium. These results and the associated pleural fluid cytology as reported by the clinical histopathologist as part of the patient’s routine clinical care are shown in **Table 2**.

**Table 2:** Shows the outcomes of the MPM primary cell culture in MPE fluid and in full medium.

Cell cultures	MPM subtype	Pleural fluid cytology at time of MPE fluid sampling	Outcome of primary cell culture	
			Cells seeded in full medium	Cells seeded in MPE fluid
MESO-392	Biphasic	Negative	Grew well and frozen at P4 (after >2 months in culture)	Culture dish confluent, cells split and transferred to full medium on day 43. Went on to becoming an established cell culture beyond P5
MESO-051	Epithelioid	Not available	Cells discarded - no attached cells on day 3	Culture dish confluent, cells split and transferred to full medium on day 38. Cells stopped growing during P1 and were discarded.
MESO-397	Epithelioid	Positive	Cells discarded - no attached cells on day 3	Cells discarded - no attached cells on day 3
MESO-398	Biphasic	Negative	Cells discarded - no attached cells on day 3	Cells discarded - no attached cells on day 3
MESO-402	Epithelioid	Positive	Grew well and frozen at P2 (after >2 months in culture)	Culture dish confluent, cells split and transferred to full medium on day 39. Went on to becoming an established cell culture beyond P5
MESO-064	Epithelioid	Positive	Grew well and frozen at P2 (after >2 months in culture)	Culture dish confluent, cells split and transferred to full medium on day 40. Went on to becoming an established cell culture beyond P5

Cell culture success with MPE fluid was similar to that in full medium. MPE, malignant pleural effusion; MPM, malignant pleural mesothelioma; P, passage.

## Discussion

Cell culture is a time-consuming process, requiring several laboratory consumables, and an artificially produced cell culture medium containing a delicate balance of nutrients required for optimal *in vitro* cell growth. Despite this, MPM cells proliferated in 100% pleural fluid, to a similar degree in both exudative and transudative MPE fluid. This indicates that it is not simply the quantity of proteins within MPE fluid that gives the fluid the biological capabilities that support cancer cell proliferation *in vitro*. Furthermore, cancer cells proliferated in heart failure non-MPE transudative pleural fluid. It is possible that the heart failure transudate pleural fluid obtained the contains growth factors since it is a filtrate of blood *via* capillaries, and therefore is also able to support cancer cell proliferation *in vitro*. However, the cancer cells might have secreted the necessary growth factors. There have been no good quality studies assessing the components of transudates in this regard.

Cheah et al. reported that non-MPM MPE fluid and benign pleural effusion fluid were associated with increased MPM cell proliferation *in vitro*, by 1.4–2.8 fold and by 1.3–2.2 fold respectively, when compared to serum-free medium as a control (8). The difference in the present study is that cells were incubated with 100% pleural fluid as compared to 30% pleural fluid in culture medium, and therefore is more representative of the clinical scenario of pleural tumor cells bathed in 100% pleural fluid as would be found in the pleural space. Importantly, our findings indicate that the biological properties of pleural fluid are not seen solely in MPM cancer cells, but also in other non-MPM cancer cells including breast and lung carcinoma. Future studies further exploring this area should therefore also focus on other malignancies as well as MPM, and we postulate that there is a likely common pathway within the pleural environment to explain these findings.

Primary cell culture for cancer cells is traditionally performed using specifically manufactured artificial cell culture medium, containing all nutrients required to provide the optimal environment *in vitro* for cell proliferation. Despite this, the results show that 100% MPM MPE fluid has the ability to support the primary cell culture of MPM cells, extracted directly from pleural fluid, without addition of any other nutrients, at a similar rate to primary MPM cell culture in full culture medium.

### Limitations

Pleural fluid viscosity limited the ability to demonstrate cell proliferation and cellular viability using the traditional ways applicable to cells cultured in clear full culture medium. To overcome this, a combination of methods were used to assess cellular proliferation, including live images, luminescence assay, and using bioinformatic algorithms to calculate relative confluence.

Cancer cells were able to survive and proliferate when seeded in 100% pleural fluid, and to our knowledge this is the first demonstration of this effect. Although the mechanism is unclear, any form of human pleural fluid (malignant, non-malignant, transudate, exudate) appears to have this effect, and this has potentially significant biological and clinical implications. If pleural fluid continuously bathing pleural cancer cells enables and sustains cell proliferation, the current management approach to MPE will need to be reconsidered. To this end, well-designed prospective clinical studies are now required to determine whether MPE fluid should be drained as early and completely as possible. Further translational studies are required to explore the biological mechanism of cancer cell proliferation when bathed in pleural fluid.

## Data Availability Statement

The original contributions presented in the study are included in the article/**Supplementary Material**. Further inquiries can be directed to the corresponding authors.

## Ethics Statement

The studies involving human participants were reviewed and approved by Oxford Radcliffe Biobank Central University Research Ethics Committee (CUREC) number: 19/A107. The patients/participants provided their written informed consent to participate in this study.

## Author Contributions

RA, NK, NR and IP conceived and designed the study. RA and NK did laboratory assays and analyzed the data. XY, MH and TD provided resources and support for the laboratory assays. RA, NR, IP, EB, RM, and MH collected samples and clinical data. RA, NK and IP analyzed clinical data. RA, NK and MD provided resources and prepared the ethics protocols. NR and IP supervised the study. PF and SM supported and facilitating the conduction of the study. All authors contributed to the article and approved the submitted version.

## Funding

This work was supported by an Oxfordshire Health Services Research Committee (OHSRC) Research Grant (ID 1247) and a Chinese Academy of Medical Sciences (CAMS) Innovation Fund for Medical Science (CIFMS, ID: 2018-I2M-2-002). NK and NR are supported by a National Institute for Health Research (NIHR) Oxford Biomedical Research Centre (BRC) grant. TD is supported by Medical Research Council, UK. XY and MH are supported by CAMS. The views expressed are those of the authors and not those of the NHS, or NIHR.

## Conflict of Interest

IP was employed by AstraZeneca in a non-related field.

The remaining authors declare that the research was conducted in the absence of any commercial or financial relationships that could be construed as a potential conflict of interest.
