# Supplementary material for: Pleural Fluid Has Pro-Growth Biological Properties Which Enable Cancer Cell Proliferation
Source: Front Oncol. 2021 Apr 28;11:658395. doi: 10.3389/fonc.2021.658395 (PMC8115017; doi:10.3389/fonc.2021.658395)
Supplement: Supplementary file 1 [file DataSheet_1.pdf]

Supplementary Material

Pleural fluid has pro-growth biological properties which enable cancer cell proliferation

Rachelle Asciak[1,2,3,*], Nikolaos I Kanellakis[1,2,4,5,*], Xuan Yao[6,7], Megat Abd Hamid[6,7], Rachel M Mercer[1], Maged Hassan[1], Eihab O Bedawi[1], Melissa Dobson[5], Peter Fsadni[3], Stephen Montefort[3], Tao Dong[6,7], Najib M Rahman[1,2,4,5,**] and, Ioannis Psallidas[1,2,**]

1. Oxford Centre for Respiratory Medicine, Churchill Hospital, Oxford University Hospitals NHS Foundation Trust, Oxford, OX3 7LE, United Kingdom
2. Laboratory of Pleural and Lung Cancer Translational Research, Nuffield Department of Medicine, University of Oxford, Oxford, OX3 7FZ, United Kingdom
3. Mater Dei Hospital, Malta
4. National Institute for Health Research Oxford Biomedical Research Centre, University of Oxford, Oxford, OX3 7LE, United Kingdom
5. Oxford Respiratory Trials Unit, Nuffield Department of Medicine, University of Oxford, Oxford, OX3 7LE, United Kingdom
6. Chinese Academy of Medical Sciences Oxford Institute, Nuffield Department of Medicine, University of Oxford, Oxford, OX3 7FZ, United Kingdom
7. MRC Human Immunology Unit, MRC Weatherall Institute of Molecular Medicine, University of Oxford, Oxford, OX3 9DS United Kingdom

*Joint first authors

**Joint senior authors

Corresponding Authors

Rachelle Asciak

Email: rachelle.asciak@ouh.nhs.uk

Tel: 0044 7922211674

Oxford University Hospitals Foundation Trust

Oxford, OX3 7LE, United Kingdom

Nikolaos I. Kanellakis PhD

nikolaos.kanellakis@ndm.ox.ac.uk

Laboratory of Pleural and Lung Cancer Translational Research,

Nuffield Department of Medicine, University of Oxford,

OX3 7FZ, United Kingdom

Supplementary methods

Full culture medium used in cell culture

The culture medium used for cell culture was Dulbecco's modified eagle medium (Sigma-Aldrich®, D5671, high (4500mg/L) glucose, without L-glutamine and sodium pyruvate, and with phenol red) with 10% foetal bovine serum (FBS) (Sigma-Aldrich® USA origin,MFCD00132239) and 100 U/ml penicillin, 100mg/ml streptomycin (full medium).

Tendency of pleural fluid to form a gel-like consistency

The pleural fluid tended to adopt a viscous gel-like consistency (Supplementary Figure), and when this happened, it was often not possible to aspirate the fluid with a P1000 pipette. This meant that a number of methods needed to be tested to determine the optimal way of demonstrating the biological effects of pleural fluid, if in fact these were present.

Supplementary Figures

Supplementary Figure 1

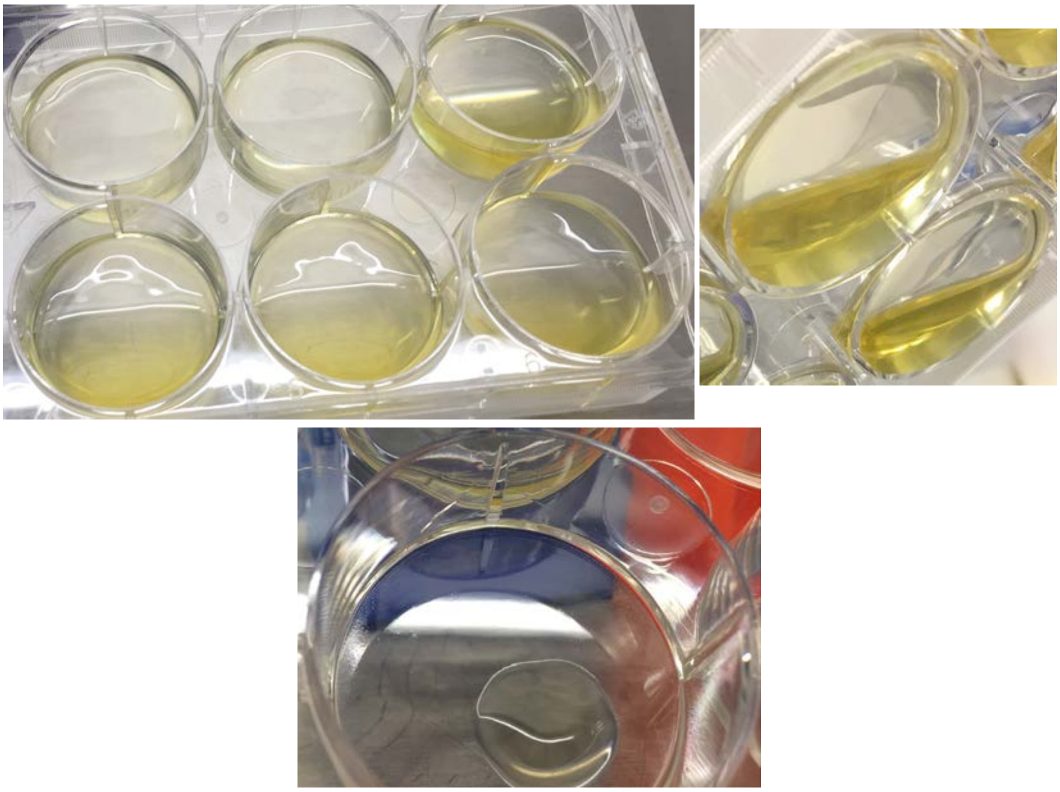

Supplementary Figure 1 shows images of wells within the 6-well plate, containing MPM cells seeded within exudate MPM MPE fluid. Tilting the plate reveals the layer of gelified pleural fluid at the bottom of the wells. Top left: the wells as viewed from above; top right: the wells as viewed from below the plate, bottom centre: a drop of pleural fluid that has adopted a gel-like consistency within the well. *MPE=malignant pleural effusion, MPM=malignant pleural mesothelioma.*

Supplementary Figure 2

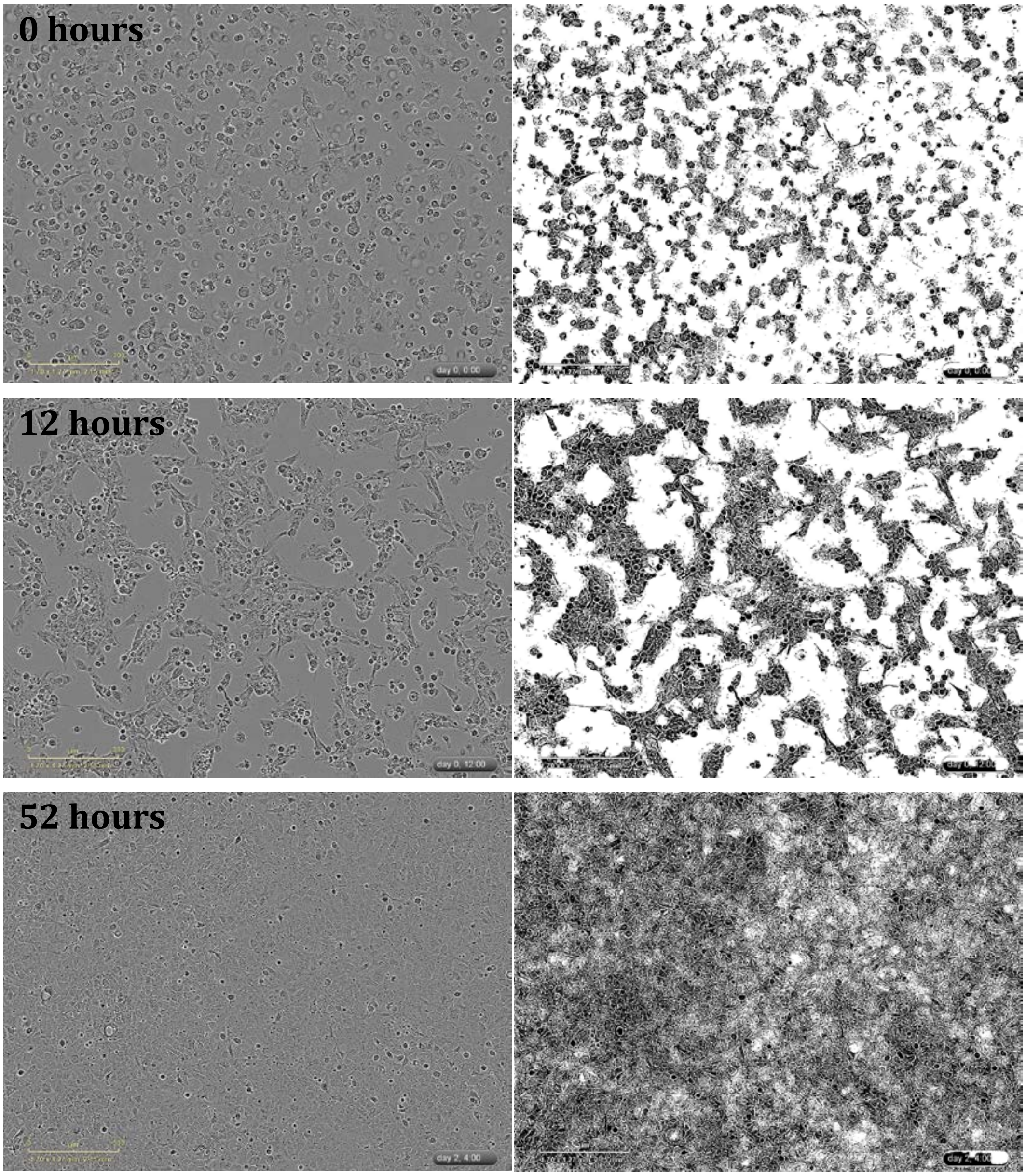

Supplementary Figure 2 shows images of MESO-024 cell culture seeded in heart failure transudate pleural fluid. The left sided images are the original images taken by Incucyte® at 10x magnification, and the right sided images are the corresponding images generated by Fiji (ImageJ) version 2.0(11) with manual adjustment of the threshold. Percentage confluence was calculated as 20.2% at seeding (top images), 29.3% at 12 hours after seeding (middle images), and 50.8% at 52 hours after seeding (bottom images).

Supplementary Figure 3

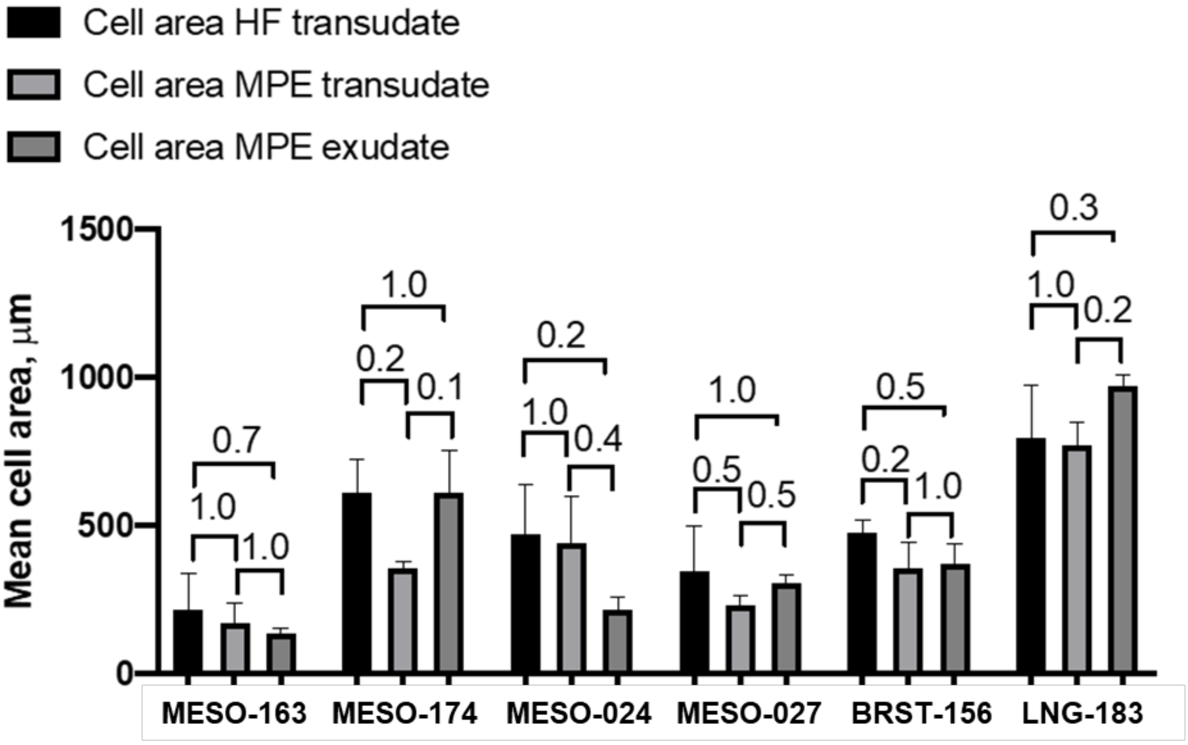

Supplementary figure 3 shows comparison between mean area of cells seeded in the different pleural fluid types. Values above the square brackets indicate the *p* values obtained (Kruskal-Wallis test) when comparing each mean, and there was no statistically significant difference between size of cells seeded in the different pleural fluid types. *HF=heart failure, MPE=malignant pleural effusion.*
